# SWI/SNF-mediated chromatin remodeling induces Z-DNA formation on a nucleosome

**DOI:** 10.1186/2045-3701-2-3

**Published:** 2012-01-20

**Authors:** Niveen Mulholland, Yan Xu, Hiroshi Sugiyama, Keji Zhao

**Affiliations:** 1 Systems Biology Center, Division of Intramural Research, National Heart, Lung and Blood Institute, NIH, Maryland, USA; 2 Department of Chemistry, Graduate School of Science, Kyoto University, Kyoto, Japan; 3 Midwest Research Institute, Maryland, USA

## Abstract

**Background:**

Z-DNA is a higher-energy, left-handed form of the double helix. A primary function of Z-DNA formation is to facilitate transcriptional initiation and activation. Sequences favoring Z-DNA formation are frequently located in promoter regions and Z-DNA is stabilized by torsional strain resulting from negative supercoiling, such as that generated by an actively transcribing polymerase or by a nucleosome remodeling event. We previously have shown that activation of the CSF1 gene by a chromatin remodeling event in the promoter results in Z-DNA formation at TG repeats within the promoter.

**Results:**

We show that remodeling of a mononucleosome by the human SWI/SNF complex results in Z-DNA formation when the DNA within the mononucleosome contains Z-DNA favoring sequence. Nuclease accessibility patterns of nucleosome core particle consisting of Z-DNA are quite different from counterpart nucleosomes containing classic B-DNA. Z-nucleosomes represent a novel mononucleosome structure.

**Conclusions:**

We present evidence that Z-DNA can form on nucleosomes though previous observations indicate the occlusion of nucleosome formation from Z-DNA.

## Background

Sequences with alternating purine and pyrimidine repeats have the highest Z-DNA forming potential [1] and are enriched in promoter regions [2]. The generation of negative supercoils behind an actively transcribing RNA polymerase [3] or by SWI/SNF-mediated nucleosome remodeling [4] can result in Z-DNA formation at promoters with TG or GC repeats. For example, TG repeats in the CSF1 gene promoter adopt a Z-DNA conformation when transcription is activated by BRG1, the ATP-dependent subunit of SWI/SNF [5]. BRG1 activity induced Z-DNA formation even in the absence of transcription indicating the torsional strain required for Z-DNA formation is generated by nucleosome remodeling rather than an actively transcribing polymerase [6]. Nucleosome mapping of the CSF1 promoter revealed that these Z-DNA forming repeats are incorporated in a nucleosome prior to BRG1 activation.

*In vitro *studies examining the interactions between Z-DNA and histones suggest that Z-DNA associates with histones but does not incorporate into a nucleosome [7]. The activity we have observed on the CSF1 promoter in vivo, prompted us to reexamine this issue. As the most basic unit of chromatin, the mononucleosome has proven to be an invaluable tool for probing the mechanisms used in transcriptional activation as well as for many other cellular functions. Since detection of Z-DNA structure *in vivo *at a genomic position associated with a nucleosome may not necessarily indicate formation of Z-DNA on the nucleosome structure because of cellular heterogeneity, we decided to address the question using *in vitro *Z-DNA formation and nucleosome assembly assays. However, the requirement for torsional strain to maintain the high energy Z-DNA helix precluded *in vitro *mononucleosome studies which use linear DNA fragments. We have combined Z-DNA stabilizing modifications with a strong nucleosome positioning sequence to assemble mononucleosomes on Z-DNA.

## Results

**Figure 1:**
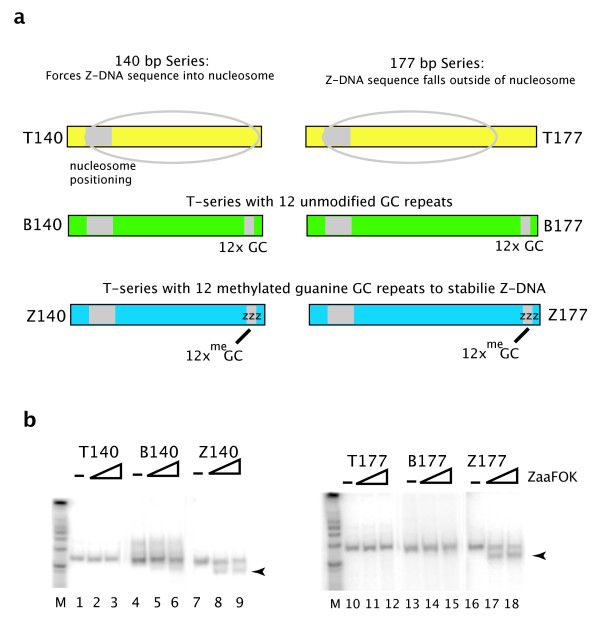
**B-DNA and Z-DNA mononucleosome templates**. a. Two series of templates were used for mononucleosome assembly: 140 bp and 177 bp. Each series consists of B-DNA without GC repeats (T140 and T177), B-DNA with GC repeats (B140 and B177) and Z-DNA (Z140 and Z177). All DNAs were generated by PCR amplification of the same parental vector. A PCR primer with GC repeats was used to generate the B-DNA-GC fragment; a primer with 8-me-guanine modified GC repeats was used to generate the Z-DNA fragment. b. Body-labeled PCR fragments described in 1a were challenged with Zaa-Fok (a fusion protein consisting of two highly specific Z-DNA binding domains, Zα, from ADAR1 and the catalytic domain of the restriction enzyme FokI) to assess Z-DNA stability. Arrow heads indicate the cleavage products.

The templates used in this study are described in Figure 1. One series of fragments are 140 bp and the other are 177 bp. Each series consist of 'T', 'B' and 'Z' fragments. The 'T' refers to TPT, the nucleosome-positioning sequence originally described by Shrader and Crothers [8]. 'B' refers to B-DNA and consists of the TPT fragment with 12 GC repeats located at the opposite end of the nucleosome positioning sequence. 'Z' refers to Z-DNA fragments which are identical in sequence to 'B' but contain 8-me-Guanine (8meG) in the GC repeats to stabilize Z-DNA structure [9]. The 8meG was incorporated in the fragment by synthesizing an oligonucleotide PCR primer with the 8meG using standard phosphoramidite chemistry [10].

The Z-DNA structure of 8meG containing GC repeats was defined on small annealed fragments [9,10]. To confirm the propagation of Z-form DNA on the larger PCR generated fragments used in this study, we used a Z-DNA specific restriction enzyme, Zaa-Fok. This recombinant enzyme consists of two copies of Zα, the Z-DNA binding domain of ADAR1, fused with the catalytic subunit of the restriction enzyme FokI [5,11]. Internally labeled PCR DNA fragments for each of the six templates were incubated with Zaa-Fok at 30°C for 30 minutes. No Zaa-Fok digestion was observed on the T fragments or the B177 fragment (Figure 1b, lanes 1-3, 10-12 and 13-15). No cleavage of the B140 fragment was observed at lower concentrations of Zaa-Fok (lanes 4-6 and 13-15) and minimal cleavage of B140 was observed at higher concentrations of Zaa-FOK (lane 6). Low levels of cutting at high Zaa-Fok concentrations may be due to either a small fraction of Z-DNA fragments in this population or by induction of Z-DNA to a fraction of templates by Zaa-binding. Importantly, both Z fragments were cut by low and high concentrations of Zaa-Fok (Figure 1b, lanes 7-9 and 16-18). Previous characterization of the properties of GC repeats with 8meG along with these results provide strong evidence for the presence of Z-DNA structure in the Z-140 and Z-177 constructs used in this study.

**Figure 2:**
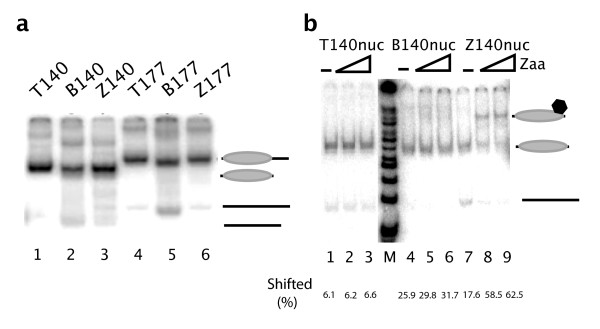
**Z-DNA incorporation in a nucleosome**. a. Body labeled PCR fragments described in Figure 1a, were assembled into nucleosomes by step salt dialysis using purified HeLa nucleosome cores. Nucleosome:DNA ratios were optimized to yield greater than 90% nucleosomal material as determined by native acrylamide gel separation of DNA from nucleosomes. All six constructs described assembled into nucleosomes at identical DNA:histone ratios. The positions of DNA and nucleosomes are indicated on the right side of the panel. b. Stability of the Z-DNA structure after assembly into a nucleosome was assayed by gel shift. Mononucleosomes assembled as in 2a, were incubated at room temperature for 30 minutes with purified Z-DNA specific binding protein Zaa. The binding reaction was run on a 4% native acrylamide gel and autoradiographed. The Z-DNA containing nucleosome assembly (Znuc) was bound by Zaa while the canonical nucleosome (Tnuc) and the GC-containing nucleosome (Bnuc) were not shifted when incubated with Zaa. The positions of shifted templates are indicated on the right side of the panel.

PCR was used to generate internally labeled DNA fragments for each of the constructs described in Figure 1a. Each DNA fragment was assembled into nucleosomes by step-down salt dialysis in the presence of HeLa nucleosome cores. The resulting materials were analyzed on native polyacrylamide gels, in a nucleosome gel-shift whereby a shift of the DNA band indicates mononucleosome formation. As shown in Figure 2a, each of the six fragments allowed for nucleosome formation. The DNA:histone ratio used for mononucleosome assembly was optimized to yield > 90% nucleosomal material; this ratio was the same for each fragment suggesting similar stoichiometry for nucleosome formation on B-DNA and Z-DNA.

Nucleosome formation may revert Z-DNA to B-DNA. To address this possibility, we performed a series of EMSAs challenging mononucleosome preparations with a Z-DNA specific binding protein, HA-Zaa, consisting of an HA purification tag fused to two repeats of the Zα Z-DNA binding domain, Zaa. Each of the six body-labeled mononucleosome species was incubated with purified HA-Zaa for 30 minutes at room temperature. Binding of HA-Zaa was monitored by native PAGE and autoradiography (Figure 2b). Neither T-nucleosome nor B-nucleosome species were bound by HA-Zaa at any protein:substrate ratio tested. Minimal binding is observed at the higher HA-Zaa concentration to the B-nucleosome; as with the results in Figure 1, this may be due either to the presence of a small fraction of Z-form material in the B-form populations or to a B- to Z-transition resulting from Zaa binding the GC repeats. Strong binding of HA-Zaa to the Z-nucleosomes was observed at all concentrations tested. We conclude from these data that Z-DNA can be incorporated in a nucleosome.

Our previous work has shown a B- to Z- transition in response to BRG1-mediated activation at the CSF1 promoter *in vivo*. Importantly, active transcription was not required for Z-DNA formation, though the extent of Z-DNA structure was greater when transcription was allowed to proceed. This raises the question as to whether chromatin remodeling by hSWI/SNF is directly responsible for Z-DNA formation. To address this question, we have used an entirely purified *in vitro *system and a restriction enzyme accessibility assay (REA).

**Figure 3:**
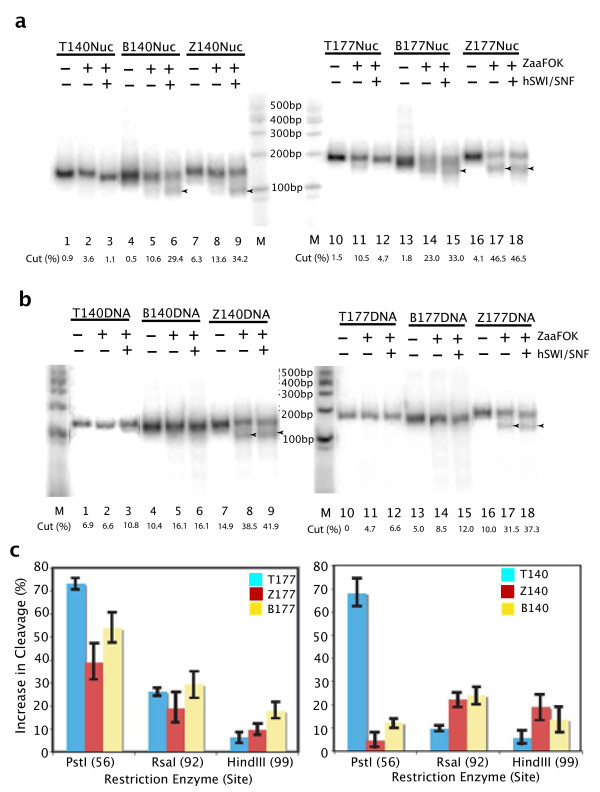
**Nucleosome remodeling by hSWI/SNF converts B-DNA to Z-DNA**. a. Mononucleosomes, assembled as described in Figure 2a, were incubated with hSWI/SNF with or without ATP for 30 minutes at 30°C. ZaaFok was added as indicated and reactions were incubated for an additional 30 minutes at room temperature. Reactions were stopped by adding Proteinase K and SDS. Cleavage by Zaa-Fok was monitored on a native polyacrylamide gel. Arrow heads indicate cleavage products. b. The affect of hSWI/SNF on Z-DNA formation on naked DNA was monitored. The conditions used in 3a. were repeated on the DNA templates. c. Restriction enzyme accessibility reveals different remodeling patterns between T140 and Z140 or B140 mononucleosomes. Mononucleomes assembled as in Fig. 2a were incubated with hSWI/SNF (with or without ATP) for 30 minutes at 30°C followed by 60 minute digestion with the indicated restriction enzymes. Cleavage bands were quantitated on native polyacrylamide gel and plotted to reflect changes in cleavage patterns due to hSWN/SNF remodeling.

Nucleosomes were assembled to yield greater than 90% nucleosomal material and incubated with hSWI/SNF. The remodeling reactions were incubated either with or without ATP for 45 minutes at 30°C, followed by 30 minutes at 30°C with Zaa-Fok. Minimal cutting by Zaa-Fok was observed after the addition of hSWI/SNF or by the ATP-activated remodeling process on either T-mononucleosomes (Figure 3a, lanes 1-3 and 10-12) or T-DNA templates (Figure 3b, lanes 1-3 and 10-12). Recognition and cutting of the Z177 nucleosomes by Zaa-Fok was not dependent on hSWI/SNF remodeling (Figure 3a, lanes 16-18). Although cleavage of Z-140 appears to be ATP-dependent (Figure 3a, lanes 7-9), we believe the inability of Zaa-Fok to cut Z140nuc in the absence of ATP is due to hSWI/SNF-nucleosome interference with Fok digestion and not due to an absence of Z-DNA. First, HA-Zaa binding was observed in the gel shift in Figure 2b and second, Z140 DNA templates were cut in the presence or absence of ATP (Figure 3b, lanes 7-9). Interestingly, the B140 and B177 mononucleosomes were cleaved by Zaa-Fok as a result of hSWI/SNF remodeling (Figure 3a, lanes 4-6 and 13-15). By contrast, neither B-form DNA template was cut by Zaa-Fok with or without ATP (Figure 3b, lanes 4-6 and 13-15). These data coupled with the observation that HA-Zaa does not bind either B-nuc construct in the EMSA analysis strongly suggests Z-DNA formation as a result of chromatin remodeling by hSWI/SNF.

The REA analysis after remodeling by hSWI/SNF resulted in different profiles for the T-nucleosome as compared with the B- or Z-nucleosomes (Figure 3c). Qualitatively similar accessibility patterns were observed between the 177-series nucleosomal templates where Z-DNA sequence is located outside of the nucleosome (Figure 3c, left panel). However, different accessibility patterns were observed between T-140 and B- or Z-140 nucleosomal templates where the Z-DNA sequence was contained within the nucleosome (Figure 3c, right panel). The changes in site accessibility resulting from nucleosome remodeling of the T-nucleosome was highest at the nucleosome positioning sequence. The PstI site, immediately 3' of the positioning sequence, yielded an expected 70% increase in accessibility upon nucleosome remodeling. Enzyme cleavage further downstream, RsaI and HindIII, was increased by less than 10% at each site. The B- and Z-nucleosome accessibility profiles showed less change in accessibility at the PstI site than the T-nucleosome but greater accessibility at the RsaI and HindIII sites. Remodeled intermediates and/or products of the B- and Z-nucleosomes appear to be different from the T-nucleosome and this difference persists throughout the nucleosome.

## Discussion

With increasing evidence for Z-DNA formation *in vivo *and for Z-DNA function, we have revisited the question of Z-DNA in the context of chromatin using tools that have become available since the first studies on the issue. The crystal structure of the B-Z transition provided evidence for the stabilization of this energetically unfavorable helix and allowed for greater understanding of how Z-DNA might exist in the context of chromosomal B-DNA [12]. Evidence in yeast suggests that increased transcriptional activity on promoters which form Z-DNA may be due to establishment of a nucleosome boundary by Z-from DNA [13]. In the present study, we describe how Z-DNA might exist in the nuclear chromatin context by providing evidence for incorporation of Z-DNA into a nucleosome. The possibility of nucleosome formation on Z-DNA also has implications in the function of Z-DNA in gene regulation. The chromatin remodeling activity of complexes such as SWI/SNF is required to activate transcription of the genes they control; and the relative density of sequences which form Z-DNA with high potential in promoter regions is high. Z-DNA formation may allow for nucleosome remodeling without ejecting the nucleosome. Since our *in vitro *nucleosome assembly assays used artificial strong nucleosome positioning sequences that may be absent in most of nucleosome formed *in vivo*, the Z-DNA-containing nucleosome structure may be less stable and thus could rapidly resolved to either B-DNA-nucleosome or Z-DNA without nucleosome by nucleosome sliding or loss of histones. Nonetheless, this would provide a window of opportunity that allows for interactions between DNA binding proteins involved in the regulation of gene expression and target sequence in the context of chromatin. The finding that nucleosome remodeling by hSWI/SNF induces the formation of Z-DNA on a nucleosome provides further evidence for a role for Z-DNA in regulating gene expression. The data provide for a potential new mechanism of action for hSWI/SNF activity at promoters with high potential Z-DNA forming sequences.

## Methods

### Mononucleosomes

T-, B- and Z-DNA templates were generated using PCR to amplify the nucleosome positioning sequence defined by Shrader and Crothers [8] from the plasmid pTPT (gift from R.E. Kingston). The primers used are: TPT-F: AAGCTGACGCGTCGGTGTTAG, TPT140-R: GGAACCTCGAGGAACCCCCTT, TPT177R: AAACGACGGCCAGTGAATTCT, B140R: TGTG(GCGCGC)_4_CCCCGATGAAAGCTTGATGTA, B177R: TGTG(GCGCGC)_4_GTGTCCCAGGGAACCTC, Z140R: TGTG(GC8meGCGC)_4_CCCCGATGAAAGCTTGATGTA, Z177R: TGTG(GC8meGCGC)_4_GTGTCCCAGGGAACCTC. Twelve GC repeats were added to the ends of primers used to generate the B-DNA series. Twelve GC repeats with internal 8-me-Guanine were added to the ends of primers used to generate the Z-DNA series. Labeled fragments were generated by incorporating γ-ATP (Amersham) into the PCR reaction. Mononucleosomes were assembled by step dialysis [14].

### Protein Purification

hSWI/SNF was purified as described [15]. Zaa and Zaa-Fok were purified as described [5].

### Gel Shift

Mononucleosomes were incubated with purified Zaa in binding buffer (12% glycerol, 20 mM HEPES pH7.9, 60 mM KCl, 1 mM EDTA, 1 mM DTT, 1 μg/10 μl dIdC, 3 μg/10 μl) in 10 μl reaction volume for 30 min at room temperature and immediately loaded on 4% native acrylamide gels.

### Nucleosome Remodeling, Zaa-Fok cleavage, and restriction enzyme accessibility

Reactions were carried out in 20 μl of reaction buffer (70 mM KCl, 15 mM HEPES [pH 7.9], 10 mM Tris-HCl [pH 7.5], 15% glycerol, 3.5 mM MgCl2, 0.3 mM DTT, 0.3 mM EDTA, 0.1 mM PMSF, BSA [20 μg/ml]) with 20 nM mononucleosome and 4 nM of hSWI-SNF for 30 min at 30°C in the presence or absence of 0.5 mM ATP. For Zaa-Fok experiments, purified Zaa-Fok (100 nM final) or buffer was added and the reaction incubated for an additional 30 min at room temperature. Stop Buffer (3% SDS, 100 mM EDTA, 50 mM Tris-HCl [pH 8.0], 25% glycerol) and 3 μl of proteinase K (20 mg/ml) was added and reactions were incubated at 55°C for 1 hour. For REA experiments, appropriate restriction enzyme was added and reactions were incubated for an additional 60 min at 30°C.

## Competing interests

The authors declare that they have no competing interests.

## Authors' contributions

NM and KZ conceived the study. NM performed the study and YX and HS provided reagents. NM and KZ wrote the paper. All authors read and approved the manuscript.
